# Gender Differences in Household Indoor Air Pollution Practices and Health Communication Preferences in Dubai

**DOI:** 10.3390/ijerph23080980

**Published:** 2026-07-28

**Authors:** Aseel A. Takshe, Ayah Abboud, Haneen Marwan Youssef, Masah Al Zuhairi Dit Amr, Marya Al Hosni, Xin Wee Chen

**Affiliations:** 1 Department of Public Health, Canadian University Dubai, Dubai P.O. Box 117781, United Arab Emirates; 20210001295@students.cud.ac.ae (A.A.); 20210001572@students.cud.ac.ae (H.M.Y.); 20210001297@students.cud.ac.ae (M.A.Z.D.A.); 20210002015@students.cud.ac.ae (M.A.H.); 2 Department of Public Health Medicine, Faculty of Medicine, Universiti Teknologi MARA, Sungai Buloh Campus, Shah Alam P.O. Box 40450, Malaysia; drchenxw@uitm.edu.my

**Keywords:** gender differences, indoor air pollution, household practices, risk perception, health communication, Dubai, United Arab Emirates

## Abstract

**Highlights:**

**Public health relevance—How does this work relate to a public health issue?**

- Indoor air pollution is an important environmental health concern in Dubai, where residents spend substantial time indoors.
- Gender differences in household practices and ventilation behaviours may influence indoor air pollution exposure-related behaviours.

**Public health significance—Why is this work of significance to public health?**

- This study provides new evidence on gender differences in indoor air pollution awareness, behaviours, and information preferences in Dubai.
- The findings identify population-specific patterns that can inform targeted environmental health communication.

**Public health implications—What are the key implications or messages for practitioners, policy makers and/or researchers in public health?**

- Indoor air quality campaigns should consider gender differences in preferred communication channels and household practices.
- Future research should examine whether these behavioural differences translate into measurable differences in indoor pollutant exposure and health outcomes.

**Abstract:**

Indoor air pollution (IAP) is an important public health issue in urban households, particularly in hot climates where residents spend substantial time indoors. While the health risks of indoor air pollution are increasingly recognised, gender differences in exposure-related behaviours, risk perception, and health communication preferences remain underexplored in Gulf cities. This study examined gender differences in awareness, household practices, and preferred information channels related to IAP among English-speaking adults in Dubai. A cross-sectional survey was conducted among 1014 adults using a structured questionnaire. Descriptive, bivariate, and multivariable regression analyses were used to examine gender-based patterns. Overall, participants showed moderate awareness of IAP; gender differences emerged across each of the four behavioural domains examined, with women more likely to use chemical-based cleaners and to open windows for ventilation. After controlling for age and education, women were significantly more likely than men to use traditional chemical-based cleaners (β = 0.69; *p* < 0.001) and to open windows for ventilation (β = 0.78; *p* < 0.001), while men more frequently relied on mechanical methods such as air filters and ceiling fans. Women were more likely to express interest in learning about IAP (adjusted OR = 2.55; 95% CI: 1.71–3.79) and to prefer community-based, informal channels such as awareness campaigns and social media. These gender differences persisted after adjustment for age and education, suggesting that indoor air quality behaviours and communication preferences may vary systematically by gender in this sample. Among this English-speaking convenience sample, gender differences were observed in household practices and preferred communication channels. These findings provide preliminary evidence that may help inform future public health communication strategies in Dubai but should not be interpreted as representative of the wider Dubai population.

## 1. Introduction

Dubai is one of the fastest-growing urban centres in the Middle East, characterised by a desert climate with extreme summer temperatures exceeding 45 °C, high humidity, and a population that spends most of its time in enclosed, air-conditioned spaces [1]. Under these conditions, indoor air quality becomes a dominant determinant of residents’ daily environmental exposure. Indoor air pollution (IAP) arises from a wide range of household sources, including cooking activities, household combustion, cleaning products, building materials, tobacco smoke, incense burning, scented products, and inadequate ventilation. These sources contribute to complex indoor pollutant mixtures that have been recognised as important determinants of respiratory and cardiovascular health [2].

Despite this burden, exposure patterns are influenced by social and behavioural factors, including gender. In many households, women are more likely to perform cleaning, cooking, and home fragrance activities that bring them into close proximity with indoor pollutants [3,4]. At the same time, men and women may differ in how they perceive indoor health risks, what ventilation strategies they adopt, and how they prefer to receive health information differences that have direct implications for public health communication and intervention design [3,5].

Research on IAP in the UAE remains limited. The available evidence suggests that many newly constructed homes in the UAE exceed guideline concentrations for formaldehyde and carbon dioxide [6], and that residents’ awareness of IAP is generally moderate [7]. However, no study in the UAE has specifically examined how gender shapes the full spectrum of household IAP-related behaviours, from product use and ventilation choices to seasonal patterns and information-seeking, within a large urban convenience sample of Dubai residents.

This study addresses that gap. Using a cross-sectional survey of 1014 adults in Dubai, we examined gender differences in awareness and risk perception of IAP; household cleaning and ventilation practices; seasonal behavioural patterns; and preferred channels for receiving health information about IAP. We additionally conducted multivariable regression analyses to determine whether gender differences in these outcomes persist after accounting for age and education, enabling stronger claims about the role of gender specifically. The findings are intended to provide preliminary evidence derived from an English-speaking convenience sample of adults living in Dubai. While they may help inform future gender-sensitive public health communication strategies, they should not be interpreted as representative of the wider Dubai population. The study is intended primarily to generate evidence and identify patterns that warrant further investigation using more representative sampling approaches. Given the limited evidence on gender-related indoor air pollution behaviours in the UAE, this study was designed primarily as an exploratory and descriptive investigation. Rather than testing a priori directional hypotheses, the study sought to characterise gender-related patterns in awareness, household practices, and information preferences and to assess whether observed differences persisted after adjustment for age and education. Accordingly, the study should be viewed as hypothesis-generating rather than hypothesis-testing, providing an empirical foundation for future representative and longitudinal investigations of indoor air pollution behaviours in the UAE.

## 2. Background and Conceptual Framework

This section reviews evidence relevant to the four behavioural domains examined in this study: cleaning product use, ventilation practices, exposure from common household sources, and health information preferences, with a specific focus on how gender shapes each domain.

### 2.1. Gendered Household Roles and IAP Exposure

Household air pollution arises when indoor pollutants accumulate beyond safe thresholds, often driven by daily activities such as cooking, cleaning, incense burning, and smoking [2,8]. Gender is a key determinant of differential exposure. Previous studies suggest that women in many cultural contexts, including parts of the Gulf region, may be more involved in domestic tasks that increase their exposure to indoor pollutants [3,4]. This greater involvement may place women in repeated contact with particulate matter, volatile organic compounds, and formaldehyde [9,10].

In Dubai, approximately 15% of residents have chronic respiratory disease such as asthma, and the combination of extreme heat, air conditioning dependence, and outdoor pollution infiltration further concentrates indoor exposures [6]. Poor housing quality, mould, and inadequate ventilation compound these risks [11]. The interaction between gender-shaped household roles and these environmental conditions creates systematically different exposure profiles for men and women, yet this has rarely been measured directly in the Gulf context.

### 2.2. Cleaning Products and Gendered Use Patterns

Cleaning products are a major source of indoor VOC exposure. Common ingredients such as glycols, ethanol, limonene, and formaldehyde-releasing preservatives volatilise readily and have been associated with eye and respiratory irritation, allergic reactions, and in chronic exposures, cancer [12,13]. Bleach-based products, when used in poorly ventilated spaces, can release chloroform and other carcinogenic compounds at measurable concentrations [14]. Green-labelled products are not necessarily safer; some emit more monoterpenes, which can react to form secondary pollutants including formaldehyde and peroxyacetyl nitrate [15].

Gendered patterns in cleaning product use are well documented. Women, who perform the majority of routine household cleaning in many societies, tend to use chemical-based cleaners that they perceive as effective and trustworthy, often shaped by familial and cultural norms [16]. Men, conversely, show higher uptake of products marketed with environmental or technology-related claims, including green-certified alternatives [17]. These differences may contribute to variation in VOC exposure within the same household.

### 2.3. Ventilation Practices and Sex Differences

Ventilation is the primary mechanism for reducing indoor pollutant concentrations. Natural ventilation through open windows or doors dilutes indoor pollutants but is constrained in Dubai’s climate by extreme summer heat and outdoor particulate pollution, meaning that during the hottest months, residents who rely solely on natural ventilation may in fact trap pollutants indoors [18]. Mechanical systems, including HVAC units, air purifiers, and exhaust fans, provide more reliable pollutant control but vary in their effectiveness depending on maintenance and filter quality [19].

Some studies have reported sex differences in ventilation preferences. Women are more likely than men to prefer natural ventilation, such as opening windows or doors, whereas men more commonly report relying on mechanical devices, such as air purifiers, ceiling fans, and exhaust ventilation [5,20]. Possible explanations include differences in thermal comfort, privacy concerns, household decision-making authority over large purchases, and familiarity with technology, household purchasing responsibilities, and decision-making authority regarding investments in air purifiers or mechanical ventilation systems [21,22]. Seasonal variation is also important: outdoor heat and safety concerns reduce window opening in summer, with potential sex differences in who reduces this behaviour the most.

### 2.4. Risk Perception and Health Information-Seeking

Gender shapes not only behaviour but also how individuals perceive and respond to environmental health risks. Women generally report higher concern about health risks associated with indoor pollutants and express greater motivation to change household practices when provided with information [3]. This heightened motivation, however, does not necessarily translate into fuller awareness, in part because of differences in the channels through which men and women access health information.

Women’s health information-seeking tends to favour informal, practical, and visually engaging formats, including social media, community campaigns, and accessible digital content [23]. Men show greater preference for technical data, product specifications, and formal or institutional sources. These divergent preferences may affect the reach of public health communication campaigns designed around a single channel or communication style. In the Gulf context, tailoring communication strategies by gender may be particularly important.

## 3. Methods

### 3.1. Study Design and Setting

This was a cross-sectional survey conducted in Dubai, UAE, between January and March 2025. Dubai was selected as the study setting because of its extreme climate, rapidly urbanising population with a diverse demographic composition, and documented gaps in IAP monitoring and public awareness [1,7].

### 3.2. Participants and Recruitment

Participants were adults aged 18 years and above who were residents of Dubai at the time of the survey and were capable of completing a questionnaire in English who gave their consent. Individuals below 18 years of age, non-residents of Dubai, and those who did not provide informed consent were excluded. Incomplete questionnaires were also excluded from analysis. Participants self-reported their gender by selecting either “male” or “female” within the questionnaire. As the questionnaire collected self-reported gender rather than biological sex, the term gender is used throughout the manuscript in accordance with the SAGER reporting recommendations. The reporting of sex and gender terminology was informed by the Sex and Gender Equity in Research (SAGER) reporting guidelines [24].

The survey was distributed via social media channels and community networks, supplemented by in-person data collection at public parks in Dubai to increase access for individuals who may not be reachable through online channels. A total of 1014 English-speaking respondents completed the survey. Recruitment through social media, community networks, and public parks may have introduced selection bias. The sample should therefore not be interpreted as representative of all Dubai residents.

### 3.3. Survey Instrument

A structured questionnaire of 24 closed-ended items was developed in English. The questionnaire was informed by a review of the literature on indoor air pollution awareness, household behaviours, ventilation practices, and environmental health communication. Items were adapted from themes identified in previous indoor air quality perception and behaviour studies and were reviewed by academic staff with expertise in public health and environmental health for content relevance and clarity. Prior to data collection, the questionnaire was pilot-tested among 25 adults residing in Dubai to evaluate comprehension and wording. Minor revisions were made based on participant feedback before final deployment. It covered: (i) sociodemographic characteristics (gender, age group, education level); (ii) familiarity with and perception of IAP; (iii) household ventilation practices (six methods, rated on a five-point scale from Never to Always); (iv) cleaning product use (three product types, five-point scale); (v) seasonal window-opening behaviour (four-point frequency scale for summer and winter); (vi) willingness to change behaviour; and (vii) interest in receiving IAP information through seven different channels (five-point interest scale). Because gender was recorded only as male or female, the analysis does not capture the experiences of gender-diverse individuals. It was selected as the primary stratification variable because of its documented relevance to household role distribution, exposure pathways, and communication preferences.

### 3.4. Statistical Analysis

Data were analysed using IBM SPSS version 29.0 and Python version 3.10.4. Descriptive statistics were computed for all variables. Gender-based comparisons for Likert-scale items were conducted using independent samples *t*-tests, and for categorical variables using Pearson chi-square tests, as reported in Table 1.

To address whether gender differences in key outcomes persisted after accounting for potential confounders, we additionally conducted multivariable analyses (Table 2). For Likert-scale outcomes (ventilation and cleaning product use, seasonal window opening, and information channel preferences), ordinary least squares (OLS) regression was used with gender (female = 1, male = 0), age (ordinal: 1 = 18–29, 2 = 30–39, 3 = 40–49, 4 = 50–59, 5 = 60+), and education level (ordinal: 1 = high school, 2 = bachelor, 3 = master, 4 = doctorate) as predictors. Age and education were selected a priori as adjustment variables because previous studies have shown that both are associated with environmental health awareness, information-seeking behaviour, and household environmental practices. For binary outcomes (interest in learning about IAP and willingness to ventilate more), binary logistic regression was used to estimate adjusted odds ratios (OR) with 95% confidence intervals. Results are presented as beta coefficients (β) for OLS models and odds ratios (OR) for logistic models, each with 95% confidence intervals and *p*-values. Although Likert-scale outcomes are ordinal, OLS regression was used to support interpretable adjusted mean differences across gender groups. Results should therefore be interpreted as adjusted differences in response scores rather than precise interval-level effects. Because multiple related outcomes were examined, findings were interpreted based on consistency across behavioural domains and effect magnitude rather than on isolated statistically significant results alone. Sensitivity analyses using ordinal logistic regression produced substantively similar results in direction and significance for all tested Likert-scale outcomes. Because the questionnaire items were analysed as individual behavioural and perception outcomes rather than combined into composite scales, internal consistency reliability statistics such as Cronbach’s alpha were not calculated. Although Likert-type responses are ordinal, OLS regression is commonly used in public health and behavioural research when response categories contain five or more ordered levels and the objective is estimation of average group differences rather than modelling latent ordinal thresholds. OLS coefficients are straightforward to interpret and facilitate comparison across multiple behavioural outcomes. To assess the robustness of this approach, ordinal logistic regression models were estimated as sensitivity analyses and produced substantively similar conclusions regarding the direction and statistical significance of gender associations.

## 4. Results

### 4.1. Sample Characteristics

A total of 1014 adults participated. The sample was roughly evenly split by gender: 521 (51.4%) female and 493 (48.6%) male. The largest age group was 18–29 years (*n* = 448, 44.2%), followed by 30–39 years (*n* = 216, 21.3%), 40–49 years (*n* = 205, 20.2%), and 50 years and older (*n* = 145, 14.3%). Most participants held a bachelor’s degree (*n* = 583, 57.5%), with smaller proportions holding high school (26.1%), master’s (12.4%), or doctoral-level (3.9%) qualifications. Education distribution differed modestly by gender (*p* = 0.016), with men more likely to hold doctoral degrees and women slightly more likely to hold bachelor’s degrees.

### 4.2. Awareness and Risk Perception

Approximately one-third of participants (32.2%) reported being “fully aware” of IAP, while half (50.1%) were “somewhat aware.” The remaining participants were neutral (13.6%) or unaware (4.0%). Men were more likely than women to report being fully aware (36.5% vs. 28.2%; *p* = 0.024). In the multivariable model, female gender was associated with slightly lower familiarity (β = −0.13; 95% CI: −0.23 to −0.03; *p* = 0.008), after controlling for age and education. However, 99.0% of women and 98.2% of men agreed that indoor air pollution can negatively affect health (*p* = 0.238), suggesting that perceived health impact did not differ by gender.

### 4.3. Cleaning Product Practices

Table 1 presents gender differences in cleaning product use. Women reported significantly more frequent use of traditional chemical-based cleaners (mean 3.3 vs. 2.6; *p* < 0.001), while men reported higher use of green-certified products (mean 3.0 vs. 2.7; *p* < 0.001). The gender differences were statistically significant, with effect sizes ranging from negligible to moderate, particularly for chemical-based cleaners and window-opening practices. Use of homemade or natural cleaners did not differ significantly by gender (*p* = 0.131). These patterns persisted in the multivariable models (Table 2): female gender was positively associated with chemical-based cleaner use (β = 0.69; 95% CI: 0.53–0.86; *p* < 0.001) and negatively associated with green-certified product use (β = −0.32; 95% CI: −0.47 to −0.16; *p* < 0.001), independently of age and education.

### 4.4. Ventilation Practices

Women reported more frequent opening of windows for ventilation (mean 3.82 vs. 3.07; *p* < 0.001), while men reported greater use of air filters (mean 3.0 vs. 2.6; *p* < 0.001) and ceiling fans (mean 2.9 vs. 2.3; *p* < 0.001). Men also reported slightly greater use of fans overall (*p* = 0.015), although the associated effect size was negligible (Cohen’s d = −0.152) and therefore this difference is not discussed further. The gender difference in window-opening behaviour was one of the largest observed across the ventilation-related outcomes, suggesting a potentially meaningful behavioural difference. No significant gender difference was found for opening doors (*p* = 0.470) or use of exhaust fans (*p* = 0.997). After adjustment for age and education (Table 2), female gender remained a strong positive predictor of window opening (β = 0.78; 95% CI: 0.62–0.94; *p* < 0.001) and a negative predictor of air filter use (β = −0.32; 95% CI: −0.48 to −0.15; *p* < 0.001) and ceiling fan use (β = −0.59; 95% CI: −0.77 to −0.41; *p* < 0.001). Education level was additionally associated with greater air filter use (β = 0.19; *p* = 0.001), suggesting that higher educational attainment independently supports mechanical ventilation uptake.

Women were also more likely to report that they would ventilate their home more often if they knew it would reduce IAP (95.0% vs. 85.2%; *p* < 0.001). In the adjusted logistic model, women had more than three times the odds of men of reporting this willingness (OR = 3.42; 95% CI: 2.14–5.46; *p* < 0.001), independently of age and education.

### 4.5. Seasonal Ventilation Behaviour

Window opening was reduced in summer (May–October) compared to winter (November–April) for both genders, consistent with Dubai’s extreme climate. In summer, men reported more frequent window opening than women (mean 2.5 vs. 2.0; *p* < 0.001). This gender difference persisted after adjustment (β = −0.46; 95% CI: −0.60 to −0.34; *p* < 0.001), suggesting that women reduce natural ventilation more than men during the hot season. In winter, gender differences in window opening were not statistically significant (*p* = 0.102).

### 4.6. Interest in Learning and Preferred Information Channels

The majority of respondents expressed interest in learning about IAP (87.4%), but this proportion was significantly higher among women (91.9% vs. 82.6%; *p* < 0.001). After adjustment, women had approximately 2.5 times the odds of expressing interest in learning (OR = 2.55; 95% CI: 1.71–3.79; *p* < 0.001). Older age was also independently associated with greater interest (OR = 1.23 per age category; *p* = 0.030), while education was not significant (*p* = 0.589).

In terms of preferred information channels, women expressed significantly greater interest in awareness campaigns (mean 3.45 vs. 2.79; *p* < 0.001), social media (mean 3.80 vs. 3.46; *p* < 0.001), online articles and blogs (mean 3.44 vs. 3.23; *p* = 0.002), and TV and radio (mean 3.38 vs. 3.23; *p* = 0.033) compared to men. In adjusted models (Table 2), female gender was the strongest predictor of preference for awareness campaigns (β = 0.69; 95% CI: 0.54–0.85; *p* < 0.001) and social media (β = 0.36; 95% CI: 0.24–0.49; *p* < 0.001). Preferences for educational seminars, printed materials, and school or university courses did not differ significantly by gender after adjustment.

## 5. Discussion

To our knowledge, few published studies have examined gender differences in household indoor air pollution practices and communication preferences in the UAE. Drawing on an English-speaking convenience sample of adults residing in Dubai, the findings identified several consistent behavioural patterns that remained evident after adjustment for age and education. Although these findings should not be interpreted as representative of the wider Dubai population, they provide preliminary evidence that may help guide future research and inform the design of more targeted public health communication strategies. The observed differences are interpreted primarily within the context of gender-related behavioural and social roles rather than biological differences between males and females. Household cleaning responsibilities, risk perception, and health communication preferences are influenced by social and cultural expectations that may differ across settings. Consequently, the findings should be interpreted as reflecting self-reported gender-related behaviours within this study population rather than intrinsic biological differences. Although the regression analyses adjusted for age and educational attainment, residual confounding remains possible. Household income, occupation, housing type, household size, smoking behaviours, frequency of air-conditioning use, and existing respiratory conditions may also influence household cleaning practices, ventilation behaviours, and perceptions of indoor air pollution. Because these variables were not comprehensively measured, the observed associations should be interpreted cautiously and may partly reflect broader socioeconomic or household characteristics. Future studies incorporating these variables would help clarify the independent contribution of gender-related behaviours. Although several comparisons reached statistical significance, many effect sizes were small according to conventional interpretation. These findings therefore indicate modest behavioural differences at the individual level. Nevertheless, the consistency of these patterns across multiple household practices and communication preferences suggests that they may still have practical relevance for designing targeted public health interventions at the population level.

### 5.1. Cleaning Behaviour and Chemical Exposure

The finding that female participants reported using chemical-based cleaners more frequently than male participants, even after adjustment for education and age (β = 0.69; *p* < 0.001), is consistent with evidence on the gendered division of household labour. Prior literature suggests that domestic cleaning responsibilities remain unequally distributed in many Arab Gulf households, where women continue to undertake a greater share of routine household labour. These culturally shaped household roles may help explain the greater reported use of chemical-based cleaners among female participants in this study, although these mechanisms were not directly measured [16,25]. In many Gulf households, routine domestic cleaning continues to be undertaken predominantly by women, even where both partners are employed. Cultural expectations surrounding caregiving and household maintenance may therefore influence not only cleaning frequency but also the choice of cleaning products and ventilation practices. These broader social roles provide one possible explanation for the behavioural differences observed in the present study, although they were not measured directly. The magnitude of this difference may be meaningful from an environmental health perspective, as these behaviours may influence exposure to indoor pollutants. The higher frequency of cleaning reported by female participants may represent behaviours associated with different opportunities for indoor air pollutant exposure; however, because indoor air quality and personal exposure were not directly measured, this interpretation remains hypothetical and warrants confirmation using objective environmental monitoring.

Conversely, men’s higher uptake of green-certified products may reflect exposure to environmental performance marketing rather than a primary concern for indoor air safety, a pattern documented by Olfat [17] in studies on gender and green consumption. Importantly, green-labelled products are not necessarily safer for indoor air quality; some release monoterpenes that generate secondary pollutants, including formaldehyde, under indoor conditions [15]. This finding suggests that public health campaigns targeting cleaning practices should not assume that promoting green products achieves gender equity in exposure reduction. Rather, campaigns targeting women specifically may consider emphasising adequate ventilation during cleaning and the risks of mixing chemical agents, as well as practical risk-reduction steps for those who most frequently perform these tasks.

### 5.2. Ventilation Strategies and Seasonal Constraints

Female participants’ strong preference for natural ventilation (window opening: β = 0.78; *p* < 0.001 after adjustment) is consistent with findings from Nordic residential settings [5] and US cooking studies [20], suggesting this is a cross-cultural pattern rather than a Dubai-specific artefact. Several explanations may contribute to these differences, including household routines, cultural practices, perceptions of comfort, and preferences regarding ventilation methods. However, these factors were not directly measured in the present study and therefore should be regarded as possible explanations rather than confirmed mechanisms [21].

The seasonal pattern is noteworthy from an environmental health perspective. During Dubai’s summer (May–October), when outdoor temperatures regularly exceed 45 °C and PM_2.5_ from dust events is elevated, female participants reported that they reduce their window opening significantly more than men (β = −0.46; *p* < 0.001). This suggests that seasonal climate constraints may reduce reliance on natural ventilation during the hottest months. The result is a seasonal pinch point: individuals who report behaviours associated with potential exposure to indoor pollutants may be less able to rely on natural ventilation. This has direct implications for potential differences in exposure-related behaviours within households.

The finding that 95% of female participants (versus 85% of men) stated they would ventilate more if they knew it reduced IAP (adjusted OR = 3.42; 95% CI: 2.14–5.46) points to an opportunity for intervention. Female participants’ strong behavioural intention suggests that they may be a receptive audience for targeted messaging, but that messaging needs to address not only motivation but also feasibility, including practical guidance on mechanical ventilation options suitable for different room types and budgets. Reduced natural ventilation may influence indoor pollutant concentrations under certain environmental conditions. However, because ventilation rates and indoor pollutant levels were not measured in this study, the observed behavioural differences should be interpreted as potential determinants of exposure rather than evidence of differential exposure itself.

An additional finding was the positive association between educational attainment and air filter use after adjustment for gender and age. This may reflect greater awareness of indoor air quality, increased access to environmental health information, or higher household purchasing capacity among more highly educated participants. Although household income was not measured, education may partially capture socioeconomic differences that influence the adoption of mechanical ventilation technologies. Future studies should examine these relationships more directly.

### 5.3. Risk Perception and the Awareness–Motivation Gap

This awareness–motivation gap is noteworthy because female participants reported lower familiarity with IAP while simultaneously expressing substantially greater interest in learning about the topic and changing related behaviours. Several explanations may account for this pattern, but the present study was not designed to identify the underlying mechanisms. The observed difference in familiarity with IAP was statistically significant but small in magnitude, indicating that male and female participants were broadly similar in their overall awareness of indoor air pollution. Future research should explore why awareness and behavioural motivation do not appear to align in the same way across genders.

### 5.4. Information Channel Preferences and Communication Design

One of the clearest gender differences observed in this study concerned information channel preferences. Female participants stated substantially higher preference for awareness campaigns (β = 0.69; *p* < 0.001) and social media (β = 0.36; *p* < 0.001), the two channels with the largest adjusted gender gaps, which provides clear direction for public health communicators. Platforms such as Instagram and TikTok, which are widely used among women in the UAE, offer opportunities for short, visually engaging, practical content on household IAP risks: when to open windows, how to choose cleaning products, and how to ventilate while cooking.

Male participants reported lower expressed interest in these channels, and the absence of significant gender differences in preferences for seminars, printed materials, or university courses, suggests that formal or institution-based communications may be more gender-neutral. Dual-channel strategies, informal digital content for women and technical or formal materials for men, may be more effective than a single-channel approach. In households where purchasing decisions are shared, messaging that frames mechanical ventilation as a family health investment may help bridge gendered preferences.

Although several information-channel differences reached statistical significance, some effect sizes were small. Consequently, these findings should be interpreted as modest preference differences rather than substantial behavioural distinctions between men and women.

Although several observed household practices have been associated with indoor air quality in previous studies, the present findings should not be interpreted as demonstrating differences in pollutant exposure or health risk between genders. Rather, they identify behavioural patterns that may influence exposure pathways and therefore provide a basis for future studies integrating behavioural surveys with objective environmental measurements.

Because of the cross-sectional design, the direction of the observed associations cannot be established. For example, participants with greater interest in indoor air quality may have adopted different household practices before participating in the survey rather than those practices leading to greater awareness. Longitudinal studies are needed to clarify the temporal relationships between awareness, household behaviours, and communication preferences.

## 6. Limitations

This study has several limitations. Its cross-sectional design precludes causal inference. The convenience sample, recruited primarily in English through online channels and public parks, may limit the generalisability of findings to the wider Dubai population and may not reflect the perspectives of all linguistic and cultural groups residing in Dubai. In particular, recruitment through English-language online platforms and public parks may have favoured younger, more highly educated, and more health-conscious individuals. Consequently, the findings should be interpreted as reflecting the characteristics of this study sample rather than those of the wider Dubai population. Future studies using probability-based or stratified sampling and multilingual recruitment strategies would improve population representativeness, including Arabic-speaking participants. All behavioural outcomes were self-reported and may therefore be subject to recall and social desirability bias; moreover, no objective indoor air quality measurements were collected. Gender was measured as a binary variable, which does not capture the experiences of gender-diverse individuals. Seasonal behaviours were assessed during a single survey period rather than through longitudinal observation. The questionnaire collected binary self-reported gender and therefore did not capture broader gender identities or distinguish gender identity from biological sex. Because multiple statistical comparisons were performed, the possibility of Type I error cannot be completely excluded. However, interpretation of the findings emphasises the consistency of behavioural patterns observed across related outcomes rather than isolated statistically significant comparisons. Finally, the questionnaire was designed to assess individual behavioural and perception outcomes rather than latent psychological constructs. Consequently, formal psychometric validation was beyond the scope of the present exploratory study. Nevertheless, future research should evaluate the questionnaire’s measurement properties in larger and more diverse populations before wider application. Future studies should seek to recruit participants using probability-based or stratified sampling strategies that better reflect Dubai’s age, socioeconomic, linguistic, and cultural diversity.

## 7. Conclusions

This study identified consistent gender-related differences in household indoor air pollution practices and communication preferences among an English-speaking convenience sample of adults residing in Dubai. These findings should be interpreted as preliminary and should not be regarded as representative of the wider Dubai population.

Female participants’ greater reliance on natural ventilation, which is severely constrained by Dubai’s summer climate, combined with their more frequent use of chemical-based cleaning products, suggests a pattern of household behaviours that may influence opportunities for indoor pollutant exposure if these behavioural differences translate into measurable differences in exposure. At the same time, females in this study demonstrate substantially higher motivation to learn about and improve indoor air quality, and a clear preference for community-based and social media channels that are underutilised in current public health campaigns in the region.

These findings support a gender-sensitive approach to IAP communication and intervention design in Dubai. The findings suggest that future public health campaigns may benefit from considering women as an important audience for indoor air pollution messaging, while recognising that these observations require confirmation in more representative population-based studies.

## Figures and Tables

**Table 1:** Participant characteristics and gender differences in indoor air pollution awareness, household practices, and communication preferences (*n* = 1014).

Variables	Overall *n* (%)	Female (*n* = 521)	Male (*n* = 493)	*p*-Value	Effect Size
**Education level**					Cramer’s V = 0.101
High school	265 (26.1)	143 (27.4)	122 (24.7)	0.016	
Bachelor	583 (57.5)	311 (59.7)	272 (55.2)		
Master	126 (12.4)	53 (10.2)	73 (14.8)		
Doctorate	40 (3.9)	14 (2.7)	26 (5.3)		
**Familiarity with IAP**				0.024	Cramer’s V = 0.097
Fully aware	327 (32.2)	147 (28.2)	180 (36.5)		
Somewhat aware	508 (50.1)	270 (51.8)	238 (48.3)		
Neutral (not sure)	138 (13.6)	79 (15.2)	59 (12.0)		
Not aware	41 (4.0)	25 (4.8)	16 (3.2)		
**IAP negatively impacts health**				0.238	Cramer’s V = 0.037
Yes	1000 (98.6)	516 (99.0)	484 (98.2)		
No	14 (1.4)	5 (1.0)	9 (1.8)		
Cleaning product use (mean, SD) *					
Chemical-based cleaners	2.9 (1.38)	3.3 (1.25)	2.6 (1.42)	<0.001	Cohen’s d = 0.517
Green-certified products	2.8 (1.26)	2.7 (1.27)	3.0 (1.23)	<0.001	Cohen’s d = −0.258
Homemade/natural cleaners	2.8 (1.26)	2.7 (1.26)	2.8 (1.25)	0.131	Cohen’s d = −0.095
Ventilation methods (mean, SD) *					
Opening windows	3.5 (1.35)	3.82 (1.06)	3.1 (1.50)	<0.001	Cohen’s d = 0.584
Opening doors	3.1 (1.20)	3.1 (1.25)	3.1 (1.15)	0.470	Cohen’s d = −0.045
Air filters	2.8 (1.37)	2.6 (1.36)	3.0 (1.36)	<0.001	Cohen’s d = −0.244
Turning on fans	3.0 (1.36)	2.9 (1.37)	3.1 (1.35)	0.015	Cohen’s d = −0.152
Exhaust fans	3.3 (1.34)	3.3 (1.38)	3.3 (1.30)	0.997	Cohen’s d = −0.000
Ceiling fans	2.6 (1.47)	2.3 (1.50)	2.9 (1.39)	<0.001	Cohen’s d = −0.393
Window opening—summer (May–Oct) ^†^	2.3 (1.10)	2.0 (1.05)	2.5 (1.11)	<0.001	Cohen’s d = −0.435
Window opening—winter (Nov–Apr) ^†^	3.0 (1.03)	3.1 (1.03)	3.0 (1.02)	0.102	Cohen’s d = 0.103
**Would ventilate more if informed**				<0.001	Cramer’s V = 0.165
Yes	915 (90.2)	495 (95.0)	420 (85.2)		
**Interested in learning about IAP**				<0.001	Cramer’s V = 0.141
Yes	886 (87.4)	479 (91.9)	407 (82.6)		
**Preferred information channels (mean, SD) ^‡^**					
Awareness campaigns	3.1 (1.32)	3.5 (1.15)	2.8 (1.40)	<0.001	Cohen’s d = 0.516
Social media	3.6 (1.02)	3.8 (1.00)	3.5 (1.03)	<0.001	Cohen’s d = 0.337
Online articles and blogs	3.3 (1.14)	3.4 (1.11)	3.2 (1.15)	0.002	Cohen’s d = 0.191
Educational seminars	3.3 (1.14)	3.4 (1.13)	3.3 (1.16)	0.116	Cohen’s d = 0.098
TV and radio	3.3 (1.14)	3.4 (1.14)	3.2 (1.14)	0.033	Cohen’s d = 0.134
Printed materials (flyers)	3.2 (1.16)	3.3 (1.16)	3.2 (1.16)	0.282	Cohen’s d = 0.068
School or university courses	3.4 (1.15)	3.4 (1.17)	3.4 (1.12)	0.572	Cohen’s d = 0.036

* Five-point scale: Never (1), Rarely (2), Sometimes (3), Often (4), Always (5). ^†^ Four-point scale: Rarely (1), ~once a day (2), ~once every few hours (3), Always (4). ^‡^ Five-point interest scale: Not at all interested (1) to Very interested (5). Means reported in the manuscript text and tables are rounded to one decimal place unless otherwise stated.

**Table 2:** Adjusted multivariable regression models for key indoor air pollution–related outcomes by gender (*n* = 1014).

Outcome	Model	Gender Coeff. (Female vs. Male)	95% CI	*p*-Value
Interest in learning about IAP	Logistic	OR = 2.55	1.71–3.79	<0.001
Would ventilate more if informed	Logistic	OR = 3.42	2.14–5.46	<0.001
Familiarity with IAP	OLS	β = −0.13	−0.23 to −0.03	0.008
Opening windows for ventilation	OLS	β = 0.78	0.62 to 0.94	<0.001
Use of air filters	OLS	β = −0.32	−0.48 to −0.15	<0.001
Use of ceiling fans	OLS	β = −0.59	−0.77 to −0.41	<0.001
Chemical-based cleaner use	OLS	β = 0.69	0.53 to 0.86	<0.001
Green-certified product use	OLS	β = −0.32	−0.47 to −0.16	<0.001
Summer window opening (May–Oct)	OLS	β = −0.46	−0.60 to −0.34	<0.001
Preference: awareness campaigns	OLS	β = 0.69	0.54 to 0.85	<0.001
Preference: social media	OLS	β = 0.36	0.24 to 0.49	<0.001

All models adjusted for age (ordinal) and education (ordinal). OR = odds ratio from binary logistic regression. β = unstandardised coefficient from OLS regression. Female coded as 1, Male coded as 0.

## Data Availability

The original contributions presented in this study are included in the article. Further inquiries can be directed to the corresponding author.
